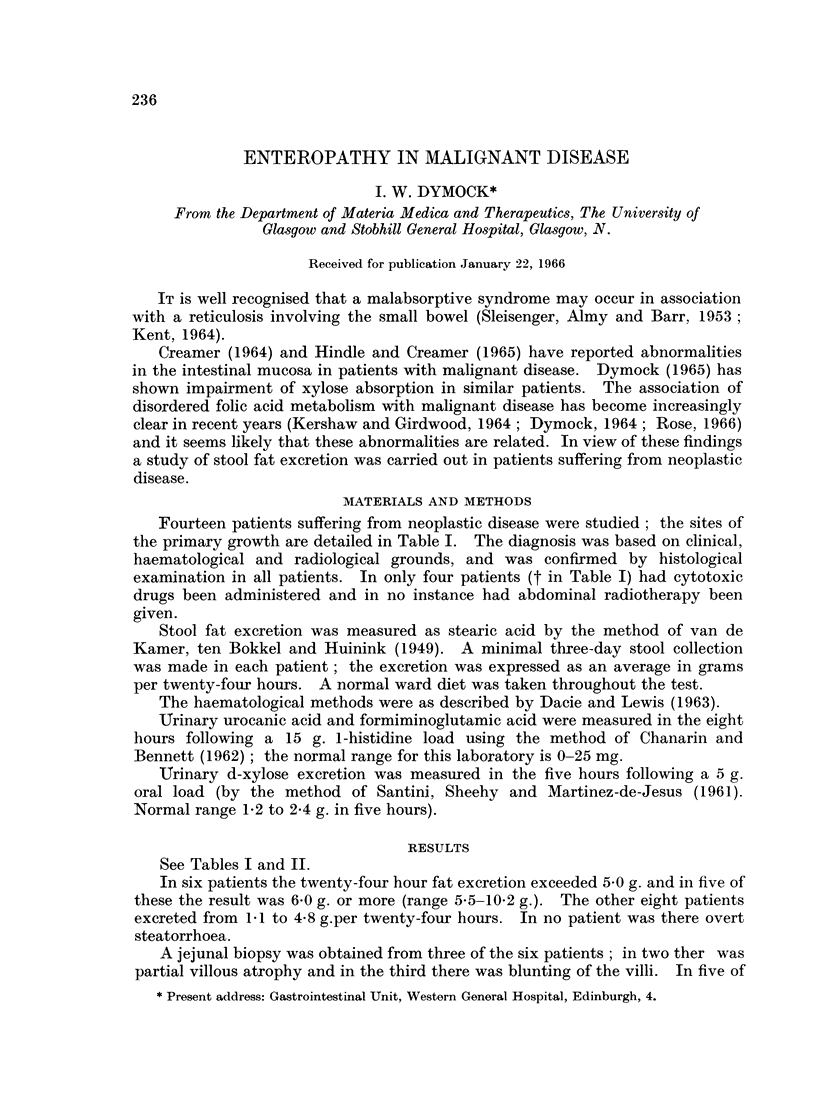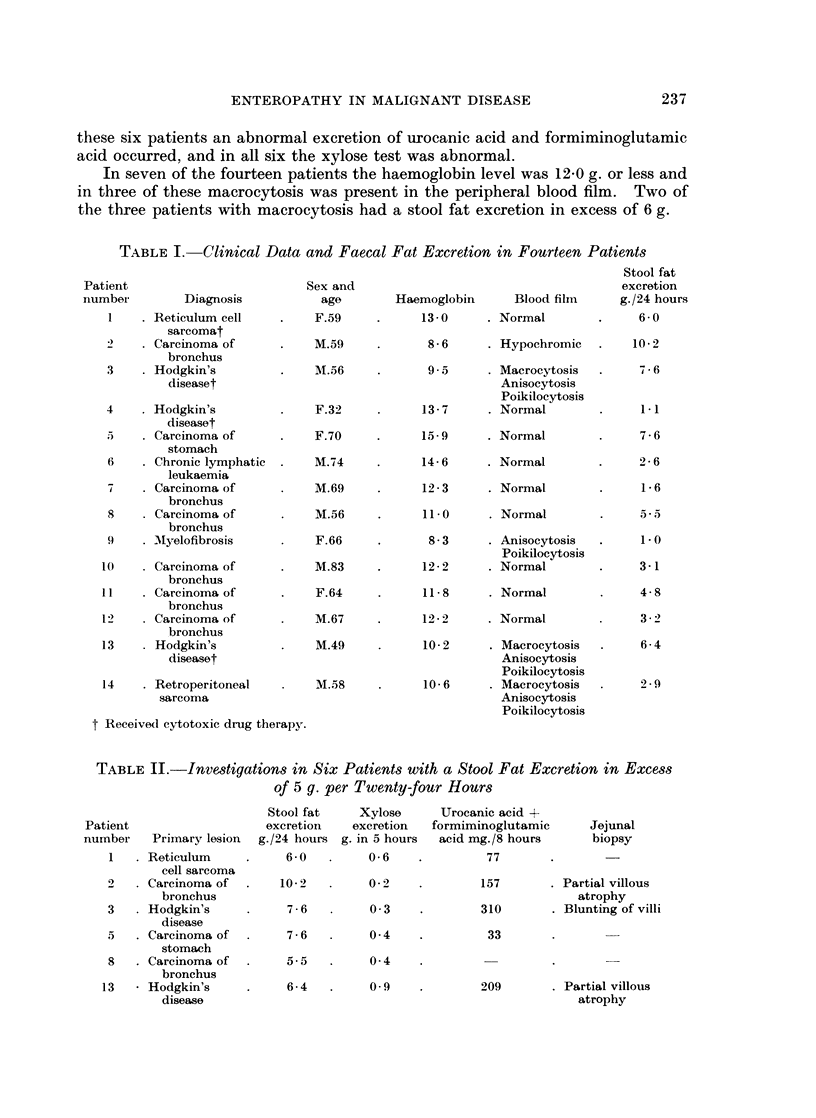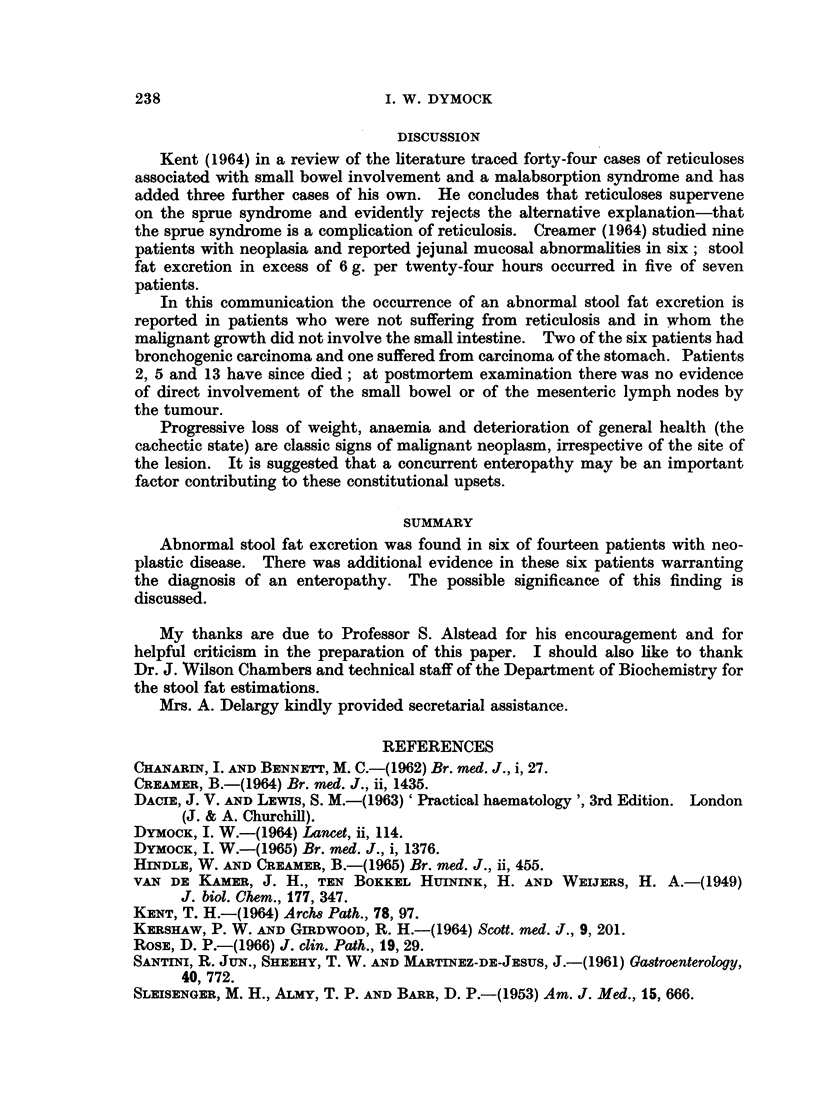# Enteropathy in malignant disease.

**DOI:** 10.1038/bjc.1966.29

**Published:** 1966-06

**Authors:** I. W. Dymock


### ENTEROPATHY IN MALIGNANT DISEASE

I. W. DYMOCK*

*From the Department of Materia Medica and Therapeutics, The University of Glasgow and Stobhill General Hospital, Glasgow, N.*

Received for publication January 22, 1966

IT is well recognised that a malabsorptive syndrome may occur in association with a reticulosis involving the small bowel (Sleisenger, Almy and Barr, 1953 ; Kent, 1964).

Creamer (1964) and Hindle and Creamer (1965) have reported abnormalities in the intestinal mucosa in patients with malignant disease. Dymock (1965) has shown impairment of xylose absorption in similar patients. The association of disordered folic acid metabolism with malignant disease has become increasingly clear in recent years (Kershaw and Girdwood, 1964 ; Dymock, 1964 ; Rose, 1966) and it seems likely that these abnormalities are related. In view of these findings a study of stool fat excretion was carried out in patients suffering from neoplastic disease.

### MATERIALS AND METHODS

Fourteen patients suffering from neoplastic disease were studied ; the sites of the primary growth are detailed in Table I. The diagnosis was based on clinical, haematological and radiological grounds, and was confirmed by histological examination in all patients. In only four patients († in Table I) had cytotoxic drugs been administered and in no instance had abdominal radiotherapy been given.

Stool fat excretion was measured as stearic acid by the method of van de Kamer, ten Bokkel and Huinink (1949). A minimal three-day stool collection was made in each patient ; the excretion was expressed as an average in grams per twenty-four hours. A normal ward diet was taken throughout the test.

The haematological methods were as described by Dacie and Lewis (1963).

Urinary urocanic acid and formiminoglutamic acid were measured in the eight hours following a 15 g. l-histidine load using the method of Chanarin and Bennett (1962) ; the normal range for this laboratory is 0–25 mg.

Urinary d-xylose excretion was measured in the five hours following a 5 g. oral load (by the method of Santini, Sheehy and Martinez-de-Jesus (1961). Normal range 1·2 to 2·4 g. in five hours).

### RESULTS

See Tables I and II.

In six patients the twenty-four hour fat excretion exceeded 5·0 g. and in five of these the result was 6·0 g. or more (range 5·5–10·2 g.). The other eight patients excreted from 1·1 to 4·8 g.per twenty-four hours. In no patient was there overt steatorrhoea.

A jejunal biopsy was obtained from three of the six patients ; in two ther was partial villous atrophy and in the third there was blunting of the villi. In five of

* Present address: Gastrointestinal Unit, Western General Hospital, Edinburgh, 4.

these six patients an abnormal excretion of urocanic acid and formiminoglutamic acid occurred, and in all six the xylose test was abnormal.

In seven of the fourteen patients the haemoglobin level was 12·0 g. or less and in three of these macrocytosis was present in the peripheral blood film. Two of the three patients with macrocytosis had a stool fat excretion in excess of 6 g.

TABLE I.—*Clinical Data and Faecal Fat Excretion in Fourteen Patients*

| Patient number | Diagnosis | Sex and age | Haemoglobin | Blood film | Stool fat excretion g./24 hours |
|---|---|---|---|---|---|
| 1 | Reticulum cell sarcoma† | F.59 | 13·0 | Normal | 6·0 |
| 2 | Carcinoma of bronchus | M.59 | 8·6 | Hypochromic | 10·2 |
| 3 | Hodgkin's disease† | M.56 | 9·5 | Macrocytosis Anisocytosis Poikilocytosis | 7·6 |
| 4 | Hodgkin's disease† | F.32 | 13·7 | Normal | 1·1 |
| 5 | Carcinoma of stomach | F.70 | 15·9 | Normal | 7·6 |
| 6 | Chronic lymphatic leukaemia | M.74 | 14·6 | Normal | 2·6 |
| 7 | Carcinoma of bronchus | M.69 | 12·3 | Normal | 1·6 |
| 8 | Carcinoma of bronchus | M.56 | 11·0 | Normal | 5·5 |
| 9 | Myelofibrosis | F.66 | 8·3 | Anisocytosis Poikilocytosis | 1·0 |
| 10 | Carcinoma of bronchus | M.83 | 12·2 | Normal | 3·1 |
| 11 | Carcinoma of bronchus | F.64 | 11·8 | Normal | 4·8 |
| 12 | Carcinoma of bronchus | M.67 | 12·2 | Normal | 3·2 |
| 13 | Hodgkin's disease† | M.49 | 10·2 | Macrocytosis Anisocytosis Poikilocytosis | 6·4 |
| 14 | Retroperitoneal sarcoma | M.58 | 10·6 | Macrocytosis Anisocytosis Poikilocytosis | 2·9 |

† Received cytotoxic drug therapy.

TABLE II.—*Investigations in Six Patients with a Stool Fat Excretion in Excess of 5 g. per Twenty-four Hours*

| Patient number | Primary lesion | Stool fat excretion g./24 hours | Xylose excretion g. in 5 hours | Urocanic acid + formiminoglutamic acid mg./8 hours | Jejunal biopsy |
|---|---|---|---|---|---|
| 1 | Reticulum cell sarcoma | 6·0 | 0·6 | 77 | — |
| 2 | Carcinoma of bronchus | 10·2 | 0·2 | 157 | Partial villous atrophy |
| 3 | Hodgkin's disease | 7·6 | 0·3 | 310 | Blunting of villi |
| 5 | Carcinoma of stomach | 7·6 | 0·4 | 33 | — |
| 8 | Carcinoma of bronchus | 5·5 | 0·4 | — | — |
| 13 | Hodgkin's disease | 6·4 | 0·9 | 209 | Partial villous atrophy |

### DISCUSSION

Kent (1964) in a review of the literature traced forty-four cases of reticuloses associated with small bowel involvement and a malabsorption syndrome and has added three further cases of his own. He concludes that reticuloses supervene on the sprue syndrome and evidently rejects the alternative explanation—that the sprue syndrome is a complication of reticulosis. Creamer (1964) studied nine patients with neoplasia and reported jejunal mucosal abnormalities in six ; stool fat excretion in excess of 6 g. per twenty-four hours occurred in five of seven patients.

In this communication the occurrence of an abnormal stool fat excretion is reported in patients who were not suffering from reticulosis and in whom the malignant growth did not involve the small intestine. Two of the six patients had bronchogenic carcinoma and one suffered from carcinoma of the stomach. Patients 2, 5 and 13 have since died ; at postmortem examination there was no evidence of direct involvement of the small bowel or of the mesenteric lymph nodes by the tumour.

Progressive loss of weight, anaemia and deterioration of general health (the cachectic state) are classic signs of malignant neoplasm, irrespective of the site of the lesion. It is suggested that a concurrent enteropathy may be an important factor contributing to these constitutional upsets.

### SUMMARY

Abnormal stool fat excretion was found in six of fourteen patients with neoplastic disease. There was additional evidence in these six patients warranting the diagnosis of an enteropathy. The possible significance of this finding is discussed.

My thanks are due to Professor S. Alstead for his encouragement and for helpful criticism in the preparation of this paper. I should also like to thank Dr. J. Wilson Chambers and technical staff of the Department of Biochemistry for the stool fat estimations.

Mrs. A. Delargy kindly provided secretarial assistance.

### REFERENCES

Chanarin, I. and Bennett, M. C.—(1962) *Br. med. J.*, i, 27.

Creamer, B.—(1964) *Br. med. J.*, ii, 1435.

Dacie, J. V. and Lewis, S. M.—(1963) ‘ Practical haematology ’, 3rd Edition. London (J. & A. Churchill).

Dymock, I. W.—(1964) *Lancet*, ii, 114.

Dymock, I. W.—(1965) *Br. med. J.*, i, 1376.

Hindle, W. and Creamer, B.—(1965) *Br. med. J.*, ii, 455.

van de Kamer, J. H., ten Bokkel Huinink, H. and Weijers, H. A.—(1949) *J. biol. Chem.*, **177**, 347.

Kent, T. H.—(1964) *Archs Path.*, **78**, 97.

Kershaw, P. W. and Girdwood, R. H.—(1964) *Scott. med. J.*, **9**, 201.

Rose, D. P.—(1966) *J. clin. Path.*, **19**, 29.

Santini, R. Jun., Sheehy, T. W. and Martinez-de-Jesus, J.—(1961) *Gastroenterology*, **40**, 772.

Sleisenger, M. H., Almy, T. P. and Barr, D. P.—(1953) *Am. J. Med.*, **15**, 666.